# An efficient procedure for plant organellar genome assembly, based on whole genome data from the 454 GS FLX sequencing platform

**DOI:** 10.1186/1746-4811-7-38

**Published:** 2011-11-29

**Authors:** Tongwu Zhang, Xiaowei Zhang, Songnian Hu, Jun Yu

**Affiliations:** 1 James D. Watson Institute of Genome Sciences, Zhejiang University, Hangzhou 31007, China; 2 Key Laboratory of Genome Sciences and Information, Beijing Institute of Genomics, Chinese Academy of Sciences, Beijing 100029, China

## Abstract

**Motivation:**

Complete organellar genome sequences (chloroplasts and mitochondria) provide valuable resources and information for studying plant molecular ecology and evolution. As high-throughput sequencing technology advances, it becomes the norm that a shotgun approach is used to obtain complete genome sequences. Therefore, to assemble organellar sequences from the whole genome, shotgun reads are inevitable. However, associated techniques are often cumbersome, time-consuming, and difficult, because true organellar DNA is difficult to separate efficiently from nuclear copies, which have been transferred to the nucleus through the course of evolution.

**Results:**

We report a new, rapid procedure for plant chloroplast and mitochondrial genome sequencing and assembly using the Roche/454 GS FLX platform. Plant cells can contain multiple copies of the organellar genomes, and there is a significant correlation between the depth of sequence reads in contigs and the number of copies of the genome. Without isolating organellar DNA from the mixture of nuclear and organellar DNA for sequencing, we retrospectively extracted assembled contigs of either chloroplast or mitochondrial sequences from the whole genome shotgun data. Moreover, the contig connection graph property of Newbler (a platform-specific sequence assembler) ensures an efficient final assembly. Using this procedure, we assembled both chloroplast and mitochondrial genomes of a resurrection plant, *Boea hygrometrica*, with high fidelity. We also present information and a minimal sequence dataset as a reference for the assembly of other plant organellar genomes.

## Background

Organellar genomes are widely used in evolutionary and population genetics studies. The plastid genome contains many essential genes, especially those required for photosynthesis. Information from multiple plastid genomes harbors suites of characters that transcend the green plant branch in the tree of life [1]. There are multiple copies of the organellar genomes in plant cells, e.g. plant leaf cells often contain 400 to 1,600 copies of the plastid genome [2]. In angiosperms, most chloroplast (cp) genomes are circular DNA molecules ranging from 120 to 160 kb. They have a quadripartite organization, consisting of two copies of inverted repeats (IRs) of 20-28 kb in size, which divides the rest of the genome into a large-single-copy region (LSC; 80-90 kb) and a small-single-copy (SSC; 16-27 kb) region [3]. Plants have larger and more complex mitochondrial (mt) genomes than other unicellular and multicellular eukaryotes. Mitochondrial genomes, especially those in seed plants, are exceptionally varied in size and structure, and their sequence contents accumulate many repetitive sequences [4,5].

Recently, there has been a dramatic increase in the number of completely sequenced organellar genomes. To date, sequences from 206 cp genomes and 47 mt genomes have been deposited in the GenBank Organelle Genome Resources. Most of them are sequences generated by the Sanger capillary sequencers [6]. With the emergence of next-generation sequencing technologies, new approaches for cp genome sequencing and assembly have been proposed because of their timesaving, high-throughput, and low-cost advantages [7-9]. As for mt genomes, three main strategies have been used: physical map-based [10-12], shotgun-based [13-15], and gene-based [16]. However, all these strategies for sequencing organellar genomes either require the isolation of cp or mt DNA from nuclear DNA [17] or are difficult to assemble because of the dynamic structure of multipartite molecules [18-20]. Isolating mitochondria and their DNA is often challenging, so that it is imperative to develop better methods for sequencing and assembling these genomes that do not include experimental sample enrichment.

In this study, we present a rapid procedure for complete cp and mt genome sequence assembly from whole genome shotgun data, without organellar DNA isolation. Using this procedure, we successfully assembled the complete cp and mt genomes of a resurrection plant, *Boea hygrometrica *(Bunge) R Br of the Gesneriaceae family. This is the first mitochondrial genome to be sequenced from a resurrection plant. *Boea hygrometrica *is an unusual, desiccation-tolerant angiosperm native to China [21,22]. Comprehensive analyses of the organellar genomes of this particular plant, and comparison with those of other plants, will help us to understand the evolution of *Boea hygrometrica*.

## Results

### Sequencing Data

We carried out four Roche/454 GS FLX sequencing runs using two fragment libraries with insert sizes ranging from 500 to 1000 bp. 4,132,392 reads were generated with a mean length of 340 bp (~1.4 Gb in total size). The quality of these reads was satisfactory, showing a read peak quality of 31 (phred quality value) and low ratio of duplicates (Table 1**and **Table 2). To validate the genome assembly, we acquired two SOLiD 4.0 runs using two mate-pair libraries (insert sizes: 1 Kb and 2 Kb). The actual insert sizes of the two libraries were 670 bp and 1,070 bp. The SOLiD dataset consists of 610,006,515 reads (30.5 Gb in total, with an average length of 50 bp). The dataset could not only be used in assembling chloroplast or mitochondrial genomes, but could also be used for future assembly of the *B. hygrometrica *nuclear genome.

**Table 1:** Data summary of Roche/454 GS FLX sequencing

Data ID	Reads number	Data (Mbp)	Mean read Length (bp)	^**a**^**Read peak quality**	^**b**^**Duplication ratio**	^**c**^**Useful ratio**
A01	713,423	255	357.2	30.4	0.07	0.96
A02	598,465	224	373.6	31.52	0.13	0.93
B01	706,828	214	302.7	30.01	0.08	0.96
B02	452,778	142	314.4	30.1	0.04	0.97
C01	653,516	239	366	31.41	0.07	0.96
C02	237,259	71	300.1	31.29	0.28	0.77
D01	506,602	157	309.7	30.37	0.06	0.97
E01	263,521	103	392.4	32.01	0.2	0.88

Total	4,132,392	1,405	339.5	30.89	0.12	0.93

^a^The means of the read qualities.

^b ^Duplicate is a group of reads whose first 50 bp sequences are identical. Duplication ratio is the number of duplicates over the total number of reads.

^c ^Useful ratio is the ratio of unique reads (reads that are different in the 50 bp sequences) and the longest reads in every duplicate over the total number of reads.

**Table 2:** Data summary of SOLiD4.0 sequencing

Library	Reads	Read length (bp)	Total data (Mbp)	^**a**^**Insert size1 (bp)**	^**b**^**Insert size 2 (bp)**
1k_F3	105,362,978	50	5,268	671	672
1k_R3	105,364,176	50	5,268		
2k_F3	199,639,680	50	9,982	1075	1065
2k_R3	199,639,681	50	9,982		

Total	610,006,515	50	30,500	\	\

^a^*B. hygrometrica *cp genome is used as the reference.

^b^*B. hygrometrica *mt genome is used as the reference

### Assembly of a Cp Genome

**Figure 1:**
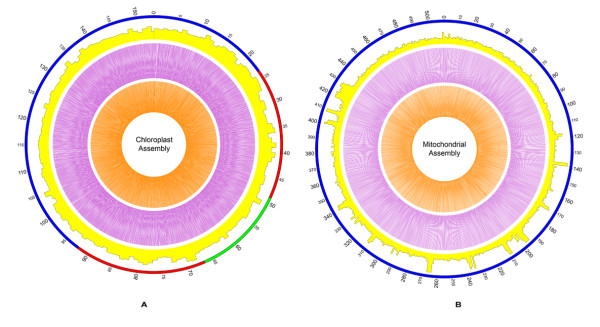
**Circular representation of an assembly of the B. hygrometrica cp and mt genomes**. Circle display (from the outside): (1) physical map scale in kilobase pairs (LSC region in blue, SSC region in green, and IRs regions in red); (2) read depths of the cp sequencing in yellow (step size: 1000 bp; cp assembly: range 0-100; mt assembly: range 0-160); (3) SOLiD mate-pair read validation with the 1-kb insert library in purple (insert size 600-1000 bp and step size 100 bp in the cp assembly and 500 bp in the mt assembly); (4) SOLiD mate-pair read validation with 2-kb library in orange (insert size 800-1200 bp and step size 100 bp in the cp assembly and 1000 bp in the mt assembly). The high variance in read depth of the mt genome results from the regions of chloroplast-like sequences. This figure was generated by the Circos program [33].

After collecting 206 plant chloroplast genome sequences, we mapped all the raw data to filter the chloroplast-like reads using Newbler (Version 2.53); 37,534 reads totaling ~13.1 Mb aligned to the chloroplast reference sequences. The reference showing the highest % alignment was *Olea europaea*, with 58.16% unique read coverage. We then used Newbler to de novo assemble all the chloroplast-like reads. This generated 502 contigs with a total length of 351,621 bp (N50 1,014 bp), the longest of which was 23,406 bp. Using perl scripts (Additional file 1) we constructed an initial contig graph for all the contigs. Only one circle graph was produced, with some mixed false links to other contigs or forks (paths with the same starting and ending contigs, but with different internal contigs). With the high copy number of the chloroplast genome, and the correlation to contig read depth, we removed contigs with lower depth and false links (read depth < 60) to choose one path with forks according to the depth in other unique contig paths. Ultimately, we obtained a chloroplast-like circle graph with a large single copy (LSC), a small single copy (SSC) and two inverted repeats (IRs) (Additional file 1). In this circle graph, there were 38 contigs of 128,041 bp in length and an N50 size of 12,185 bp (counting IR regions as one copy). The read depth in the LSC or SSC regions was nearly half of the read depth in the IR regions, because Newbler assembled reads in different IRs copies as a single contig. There are almost no gaps between connected contigs, except for a gap base "C" between contig00004 and contig00163 and an insert base "G" between contig00002 and contig00446. Most zero base gaps among contigs are logical according to the contig graph principle from Newbler (see the user's manual of the Roche 454 Newbler software). After we corrected the contig assembly and checked the connection among contigs in the circle graph (Figure 1A), the assembly became a single circle: the complete *B. hygrometrica *chloroplast genome, which is 153,493 bp and has a GC content of 37.59%.

### Assembly of an Mt Genome

In comparison to non-plant unicellular and multicellular eukaryotes, plants have larger and more complex mitochondrial genomes [23]. All the features of plant mt genomes, including RNA editing, genomic recombination, trans-splicing, and insertions of "foreign" DNA from other genomes [24] make assembling mt genomes difficult. As recent studies have shown, genome sequences vary exceptionally in size, structure, and sequence content, especially among seed plants [4,5]. However, there are essential genes that are highly conserved in almost all plant mt genomes, such as NADH dehydrogenase, succinate dehydrogenase, ubichinol cytochrome c reductase, cytochrome c oxidase, and ATP synthase. Using these genes, we could identify assembled contigs originated from the mt genome. Such gene-based procedures have been used to enrich plant mtDNA for mt genomic sequencing [16].

**Figure 2:**
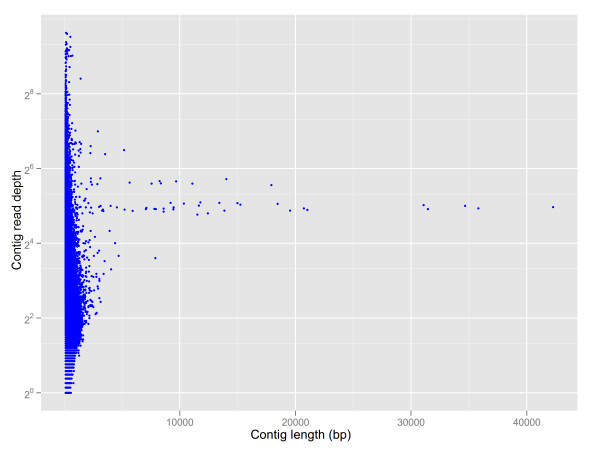
**Contig length and read depth distribution in a de novo assembly of raw data**. The Y-axis is an exponential scale with powers of 2.

Our procedure for assembling mt genome was as follows. First, we assembled all raw reads with Newbler (Version 2.53). There were 231,227 contigs with a total length of ~71 Mb. The contig N50 value was 386 bp, and the longest length was 42,272 bp. There were some contigs with length > 5 kb and high read depth, which were separated with other, shorter contigs (Figure 2). Second, as in the cp contig graph assembly, we filtered out mt contigs that included essential mt genes, and constructed an initial mt contig graph with perl scripts. Third, although there were some cp-like contigs mixed in the graph, we were able to remove the full-path cp contigs from the graph after aligning all contigs to the cp genome. Contigs that were partial in a path, but were cp-like, were also saved for further analysis, because fragments of the cp genome are frequently transferred to the mt genome [25]. False links and forks were removed according to read depths of the contigs (20 < read depth < 60). Fourth, we obtained a revised graph with three repetitive contigs (Additional file 1). In the circle graph, there were 71 contigs totaling 507,999 bp and with an N50 size of 18,440 bp (counting redundant contigs as one). To correct the position of the repetitive contigs and to construct the master circle, we mapped all SOLiD mate-pair data to the contigs that spanned repetitive contigs, using the SOLiD BioScope software. Analyzing gap-spanning repetitive contigs, we obtained major and minor links (according to the number of mate-pair reads that were mapped to both end contigs of the repetitive contigs). The major links became the last master circle (Table 3). Ultimately, we again used all raw reads from both Roche/454 and SOLiD platforms to fill the remaining gaps and remapped the last master circle for read depth distribution and mate-pair read connections (Figure 1B). The master circle of *B. hygrometrica *mitochondrial genome is 510,519 bp and has a GC content of 43.27%.

**Table 3:** SOLiD mate-pair read links of spanning repetitive contigs

	*Links*	*5'-Contig*	*3'-Contig*
Repeat 1	**3990**	**Contig00011**	**Contig00012**
	**3226**	**Contig00059**	**Contig00050**
	206	Contig00012	Contig00050
	210	Contig00011	Contig00059
Repeat 2	**810**	**Contig00001**	**Contig00026**
	**615**	**Contig00004**	**Contig00032**
	333	Contig00001	Contig00004
	186	Contig00026	Contig00032
Repeat 3	**53**	**Contig00026**	**Contig00281**
	**51**	**Contig00395**	**Contig200056**
	31	Contig00026	Contig00395
	14	Contig00281	Contig200056

The contigs linked to the master circle are shown in bold.

### Minimal Sequencing Data for Organellar Genome Assembly

After finishing the organellar genome assembly for *B. hygrometrica*, we carried out a simulation study to determine a minimal sequencing dataset for our procedure. We randomly sampled 50-1,400 Mbp sequences from the raw Roche/454 data, and assembled the organellar genomes with our procedure. Flow-cytometry study showed that the genome size of *B. hygrometrica *is about 300 Mbp, which is twice large as that of *Arabidopsis thaliana *(our unpublished data). The sequencing coverage of *B. hygrometrica *is about 4.68×.

The result of the simulation showed that the minimal sequencing data required for a complete assembly is about 300 Mbp (coverage 1×) for the cp genome, and 500 Mbp (coverage 1.7×) for the mt genome (Table 4); the latter is just one machine run of the Roche/454 platform. The minimal sequencing data may be different in other plant species, as the copy number of chloroplast or mitochondrial genomes in all plant cells may vary significantly [2,26]. For example, photosynthetic eukaryotes maintain 50-100 copies of their chloroplast genomes per chloroplast. However, there can be up to 250-500 genome copies per chloroplast [27]. Therefore, nuclear genome size is not an essential factor to the minimal sequencing data. In fact, plants with large mt genomes may not need more sequencing data than those with small mt genomes. The minimal sequencing data are not only important for compete assembly of plant organellar genomes, but also provide relevant data for the nuclear genome sequencing effort.

**Table 4:** Analysis of minimal sequencing coverage for the complete organellar genome assembly of *B. hygrometrica*

Sample data (Mbp)	Sequencing coverage (X)*	cpDNA coverage (%)	mtDNA coverage (%)
50	0.17	47.32	9.05
100	0.33	88.08	43.77
200	0.67	98.90	88.72
400	1.33	100.00	99.82
600	2.00	100.00	100.00
800	2.67	100.00	100.00
1000	3.33	100.00	100.00
1200	4.00	100.00	100.00
1400	4.67	100.00	100.00

*The flow cytometry study showed that the genome size of *B. hygrometrica *is about twice that of *Arabidopsis thaliana *(~300 Mbp).

## Discussion

**Figure 3:**
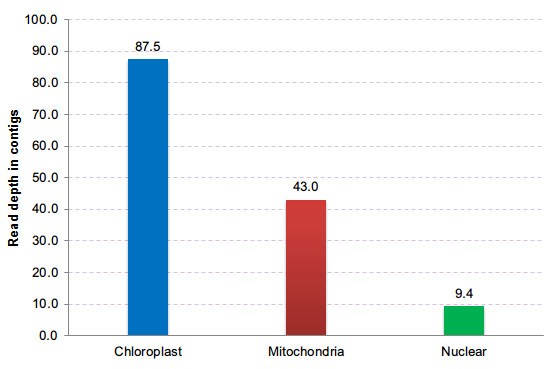
**Average read depth of contigs in chloroplast, mitochondrial, and nuclear DNAs**.

We presented a novel, rapid procedure for assembling organellar genome sequences, which take advantage of shotgun sequencing protocols and eliminates cumbersome steps, such as isolation of organellar DNAs, as compared to other sequencing strategies. Two recent studies have been published that report the sequence of chloroplast genomes from total genomic DNA based on the SOLiD [28] and Illumina platforms [29]. However, compared to these two methods, our procedure, based on the 454 sequencing platform, is superior because of the longer sequencing reads and the efficient assembling software (Newbler), which enabled the complete assembly of the organellar genome without reference sequences or gap-filling experiments. Considering the repeats in the organellar genome, the procedure required other long segment PCR experiments or long mate-pair library data (such as SOLiD sequencing) to resolve the repeats, especially in the mitochondrial genome. As part of our assembly procedure, the read-depth of contigs is important for separating chloroplast or mitochondrial genomic contigs from nuclear contigs **(**Figure 3). The substantial coverage biases across the organellar genome are also present in the 454 sequencing platform, as have been found for the Illumina sequencing platform [30]. However, comparing the different coverage depths between the organellar and nuclear genome assemblies, there are substantial coverage biases across organellar genomes; and these can be ignored if the total data are adequate for assembling organellar genomes. The read-depth of contigs belonging to chloroplast or mitochondrial genomes depends on their copy numbers in the cell and their proportion of the total DNA. The copy number of plant organelles is difficult to estimate. Therefore, the lowest sequencing coverage used to complete the assembly of an organellar genome depends on the plant (nuclear) genome size and the plant materials used for sequencing. The larger the copy number in a plant cell (such as in fresh leaf), the less sequencing data is needed. Moreover, the copy number difference between organellar and nuclear DNA is independent of the sequencing platform. Therefore, this procedure can be extended to other platforms with low coverage genome sequencing, such as the Illumina HiSeq platform. In addition, our strategy is also very useful for plant sequencing projects when an adequate coverage has not been reached, but a data quality assessment is required. For example, 454 sequencing data from a single lane or less may be enough for organelle-rich samples, and thus the cost for such a data acquisition becomes reasonable. Finally, there are new low-throughput sequencing platforms already in the market, such as IonTorrent and 454 GS Junior, for which our procedure is appropriate for data evaluation.

## Conclusions

We have successfully applied a new, efficient procedure to determine the complete chloroplast and mitochondrial genome sequences of the resurrection plant, *Boea hygrometrica*. Subsequently, we have also applied this approach to completely assemble the mt genome of *Phoenix dactylifera L *with only one run of Roche/454 data, and two Hassawi rice (*Oryza sativa L*. in Saudi Arabia) organellar genomes (both cp and mt genomes) (data not shown). Therefore, we are confident that our efficient and straightforward procedure will prove useful for further organellar genome sequencing and assembly.

## Materials and methods

### Materials and datasets

*Boea hygrometrica *plants were collected from their natural habitat in Beijing, and maintained in a greenhouse (approximately 25°C, 16 h/8 h light period) with regular irrigation. After 2 weeks of growth, fresh green leaves were collected. We extracted genomic DNA from 50 g of leaves according to a CTAB-based protocol [31]. According to the manufacturer's manual for the 454 GS FLX Titanium, we used 5 μg of purified DNA to construct the libraries. In addition, two mate-pair libraries were constructed for the SOLiD 4.0 (Applied Biosystems, Foster City, CA) sequencing platform. We downloaded 206 sequenced plant chloroplast genome sequences from the NCBI (National Center for Biotechnology Information) ftp site http://ftp.ncbi.nih.gov/genomes/Chloroplasts/plastids and 47 sequenced plant mitochondrial genome sequences from NCBI Organelle Genome Resources http://www.ncbi.nlm.nih.gov/genomes/GenomesGroup.cgi?taxid=33090&opt=organelle.

### Assembly pipeline

**Figure 4:**
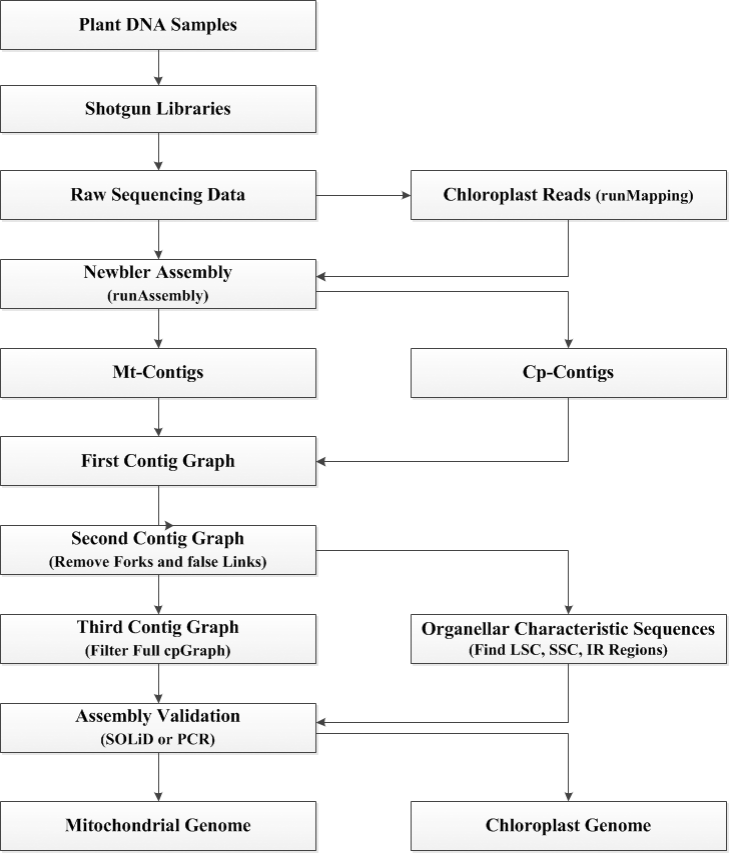
**Pipeline for plant organellar genome assembly**. Mt-contigs and cp-contigs include genes that are highly conserved among all plants. The conserved parts of sequence contigs can be identified first, and using SOLiD mate-pair libraries or PCR walks, we can confirm putative connections and close gaps among contigs empirically.

Our organellar genome assembly pipeline is shown schematically in Figure 4. Unlike other protocols [10,16,17], it does not require the isolation of organellar DNAs from the total DNA. The total shotgun sequence reads contain a mixture of sequences from both organellar and nuclear genomes. For the Roche/454 GS FLX platform, the nuclear genomic data with a low average coverage is not sufficient for alignment of long contigs with a random DNA library. For a reference genome sequence dataset, there is a high correlation between contig read depth and the number of copies in the genome [32]. Per contig read depth analysis of assemblies based on 454 reads therefore enables de novo detection of high-copy chloroplast or mitochondrial contigs. According to the manual of the Roche/454 sequencing assembly software Newbler, contigs are constructed with the trimmed reads and there are almost no overlaps among contig sequences. Moreover, a file named "454AllContigGraph.txt" in the results of Newbler assembly records all the contigs read depths, and the relatedness of contig connections, which can be used to build a contig graph. In this contig graph, contigs are the nodes, and reads spanning between them (starting in one contig and continuing or ending in another) are the edges. The initial contig graph of organellar genomes are mixed with other repeat-containing nuclear contigs. Taking the advantage of the difference of read depths among contigs, we could isolate the organellar contigs from the nuclear contigs. In this procedure, for the organellar genome assembly of *Boea hygrometrica*, the average read depths of contigs were 87, 43, and 9 in the chloroplast, mitochondria, and nuclear assemblies, respectively (Figure 3). Therefore, we set the coverage to 60 and 20 to separate contigs belonging to the three different genomes, except for repeat-containing contigs. The false links and forks in the assembly graph normally belong to different genomes. Therefore, they can be removed in the same way. After removing contaminating nuclear contigs, only clean and complete organellar genome graphs should remain. We could subsequently use all the raw data to fill the gaps between two connected contigs. Most of the gaps between two connected contigs are zero or one mismatch base pair (see the manual of Roche 454 Newbler software). To validate the final genome assembly, we need to incorporate other types of data or experiments to ensure the correct connections among contigs, such as SOLiD mate-pair data or PCR walks.

### Accession numbers

The genome data have been submitted to the National Center for Biotechnology Information (NCBI) database. The accession numbers are [GenBank: JN107811] and [GenBank: JN107812] for *Boea hygrometrica *chloroplast and mitochondrial genomes, respectively.

## Competing interests

The authors declare that they have no competing interests.

## Authors' contributions

TZ was responsible for developing the procedure and drafting manuscript. XZ helped with the design of the study and performed 454 sequencing. SH and JY supervised the project and revised the manuscript. All authors read and approved the final manuscript.

## Supplementary Material

Additional file 1 **Includes the contig graphs of both cp and mt genome assemblies of *B. hygrometrica*, and perl script information for this procedure**. Click here for file
