# Supplementary material for: An efficient procedure for plant organellar genome assembly, based on whole genome data from the 454 GS FLX sequencing platform
Source: Plant Methods. 2011 Nov 29;7:38. doi: 10.1186/1746-4811-7-38 (PMC3248859; doi:10.1186/1746-4811-7-38)
Supplement: Additional file 1 — Includes the contig graphs of both cp and mt genome assemblies of B. hygrometrica, and perl script information for this procedure. [file 1746-4811-7-38-S1.DOC]

PLANT METHODS

An efficient procedure for organellar genome assembly using whole genome data form 454 GS FLX sequencing platform

**Tongwu Zhang^1, 2^, Xiaowei Zhang^2^, Songnian Hu^2^, and Jun Yu^2,*^**

^1^James D. Watson Institute of Genome Sciences, Zhejiang University, Hangzhou 31007, China

^2^Key Laboratory of Genome Sciences and Information, Beijing Institute of Genomics, Chinese Academy of Sciences, Beijing 100029, China

**SUPPLEMENTAL MATERIAL**

**Supp. Fig. S1.** A De novo cp assembly from a single region of cp-like Roche/454 reads. The large single copy, the small single copy, and the inverted repeats are shown in blue, green, and red, respectively. The boxes stand for contigs and the lines indicate the link (overlapping) between two contigs. The numbers in the boxes show contig name, length, and read depth. The numbers on lines are reads spanning two contigs. This figure was generated with Graphviz (<http://www.graphviz.org/>).


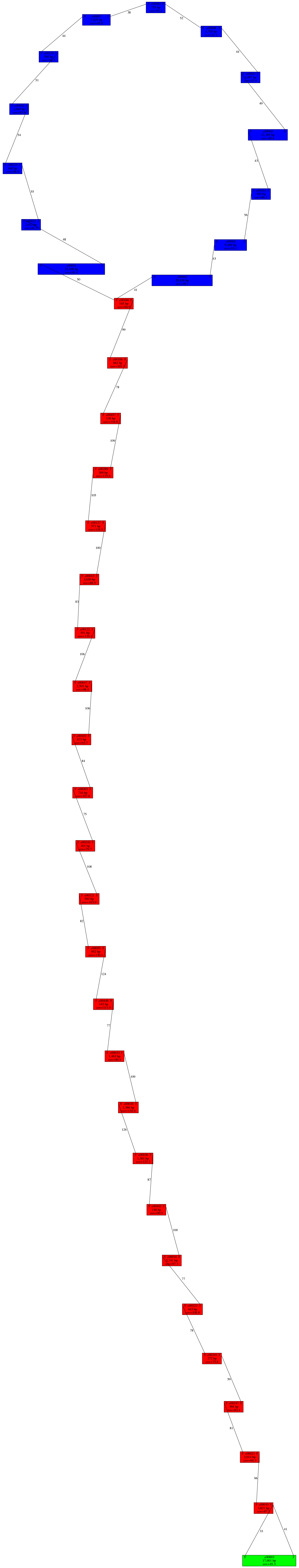


**Supp. Fig. S2.** *De novo* mt sequence assembly from total sequencing reads. The boxes stand for sequence contigs and the lines indicate the overlapping between two contigs. The numbers in the boxes show contig names, lengths, and read depths. The numbers on the lines are the number of reads spanning two contigs. The boxes in green show contigs with matched mt genes, and the red boxes show three repeat contigs. The figure was generated with Graphviz (<http://www.graphviz.org/>).


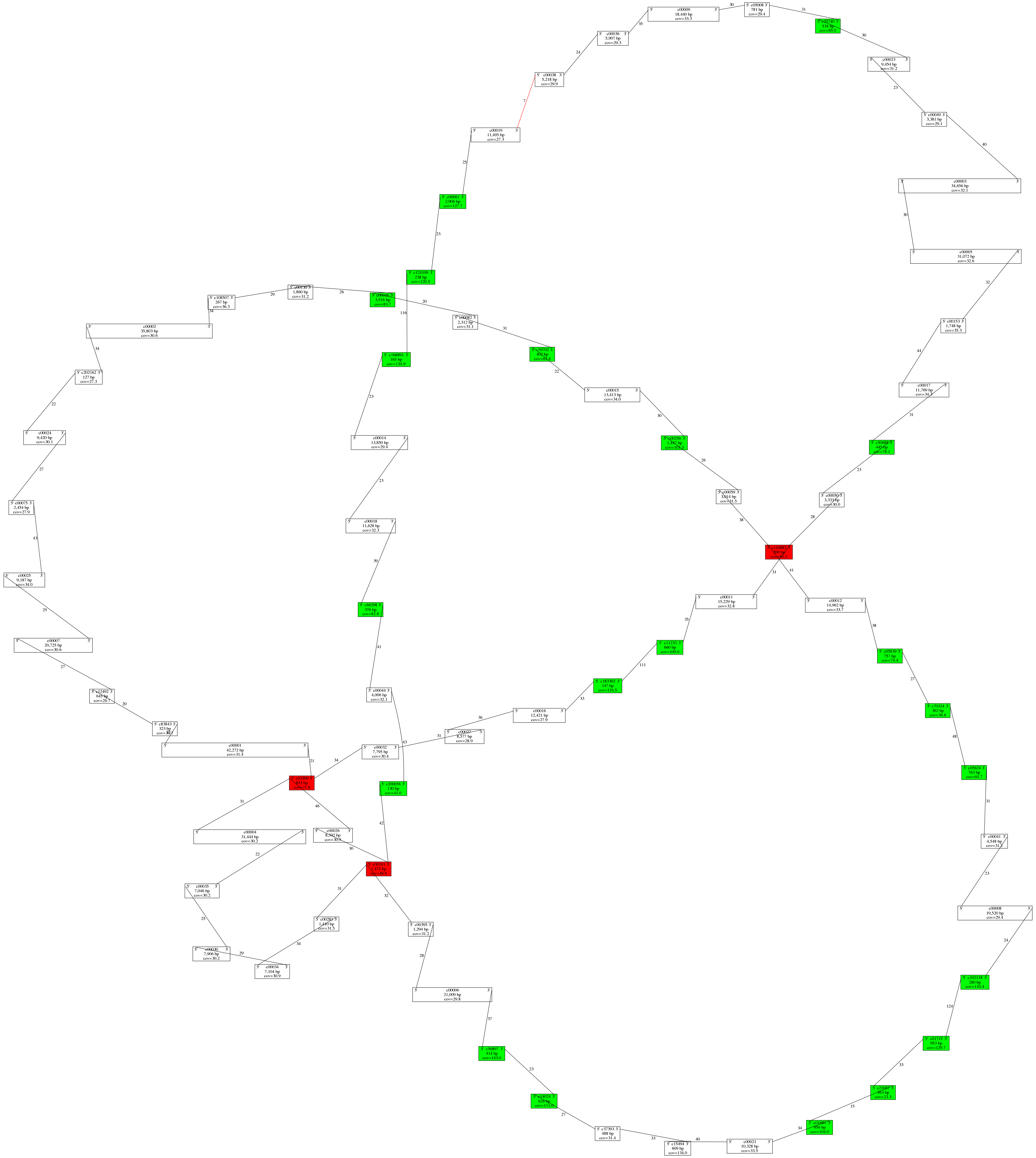


**Perl scripts**:

Two mainly perl scripts used in this procedure is bb.454contignet and bb.fastareorder, which can be download from the laboratory of Philipp W. Simon, USDA-ARS Vegetable Crops Research Unit (<http://www.vcru.wisc.edu/simonlab/sdata/software/>), here, we thanks Douglas Senalik ([dsenalik@wisc.edu](mailto:dsenalik@wisc.edu)) and Simon Gladman ([simon.gladman@csiro.au](mailto:simon.gladman@csiro.au)) for sharing those two perl scripts.
